# Data on growth performance of marine *Chlorella* sp. cultured in different cost-effective media

**DOI:** 10.1016/j.dib.2023.109894

**Published:** 2023-12-01

**Authors:** Trina Das, Sifatun Nur, Mohammad Ekramul Haque, Mahima Ranjan Acharjee, Subeda Newase, Sadia Afrin, Helena Khatoon

**Affiliations:** Department of Aquaculture, Chattogram Veterinary and Animal Sciences University, Chattogram 4225, Bangladesh

**Keywords:** Growth curve, *Chlorella* sp., Cost-effective media, Cow dung

## Abstract

This paper presents the data on growth performance of marine *Chlorella* sp. cultured in different cost-effective media including cow dung, cow urine, poultry litter, compost, NPK (nitrogen, phosphorus, and potassium), and UTR (Urea, TSP, and red potash). Growth curve of *Chlorella* sp. was determined at 5 mg of cow dung, poultry litter, compost, NPK, UTR and 5 µL of cow urine per 350 ml sea water (25 ppt) to identify the onset of stationary phase. Further four media among these were selected to continue the experiment at 8 mg and 11 mg of concentration. The higher cell densities were 4.21 × 10^6^ and 4.18 × 10^6^ cells/mL for NPK at 8 mg and 11 mg of concentration on 6th and 5th day, respectively. Cow dung with an 11 mg of concentration exhibited 2.67 × 10^6^ cells/mL on the 3rd day, which is around 1.5 times greater than the highest growth in the same concentration of poultry litter. *Chlorella* sp. had a higher cell density in NPK media than in other media, however it was discarded since it is inorganic and costly. Due to the low cell density in cow urine media and the prolonged stationary phase in poultry litter media, the focus of the subsequent study was then placed on cow dung media. The data will contribute to the selection of locally available and cost-effective culture media by determining the stationary phases for specific microalgal species which will replace the costly and labor-intensive commercial media.

Specifications Table

**Table:** 

Subject	Food Science, Aquatic Science
More specific subject area	Effect of different organic and inorganic media on the growth of *Chlorella* sp.
Data format	Raw and analyzed primary data
Type of data	Table and graph
Data collection	Growth performance was analyzed by counting cell density using neubauer haemocytometer. The media utilized to grow microalgae included cow dung, cow urine, poultry litter, compost, NPK (nitrogen, phosphorus, and potassium), and UTR (Urea, TSP, and red potash) media. A control against these was conway culture medium. *Chlorella* sp. was cultured in triplicates for each medium. The acquired data were further analyzed through MS Excel.
Data source location	Disease and Microbiology lab, Department of Aquaculture, Faculty of Fisheries, Chattogram Veterinary and Animal Sciences University, Khulshi-4225, Chattogram, Bangladesh
Data accessibility	Data are available with this article and also at Repository name: Mendeley Data Data identification number: DOI:10.17632/jw6sr2k3df.1 Direct URL to data: https://data.mendeley.com/datasets/wzcxws83wb/1

## 1 Value of the Data


 

• The data will contribute to the selection of potential culture media for specific microalgal species.
• The data describes the use of locally available and cost effective organic and inorganic media to culture Chlorella sp. instead of costly and labor-intensive commercial media.
• This technology will also be convenient and easy to adopt for farmers and those engaged in the large-scale production of Chlorella sp.
• Marine fish and shellfish can be fed with live microalgae or microalgae-formulated feed generated through large-scale production.

## 2 Background

Microalgae are widely used in aquaculture as live feed for larvae culture industry and premix for feed formulation, and widely used in shrimp hatcheries. Most marine invertebrates depend on microalgae for their whole life cycle. Microalgae must be readily available in sufficient quantities to be used as fish and shrimp food. Mass cultures of microalgae are required to supply that demand. Several commercial media have been used for mass cultivation of microalgae because they offer the nutrients required for their growth. These media are relatively expensive. One such medium is conway medium. Pure cultures of *Chlorella* sp. were cultivated using the standard cultivation protocol in conway medium [3]. Furthermore, *Chlorella* sp. has been produced on an industrial and laboratory scale, but no information has been found indicating that farmers culture this species in farm level. The present requirement is for readily available and affordable media to reduce the production cost. *Chlorella vulgaris* was cultured in different concentrations of panchagavya in India [4]. A mixture of five cow products—milk, curd, ghee, dung, and urine—is known as panchagavyam (pancha = five, gavyam = product of cow). Panchagavyam is the final product of fermenting these ingredients in accordance with the specifications [2]. However, no study has been done in Bangladesh to develop alternative and cost-effective media for microalgae culture (Table 1).

**Table 1:** Nitrogen content (%) of different media.

Treatment	Total Nitrogen Content (TN) (%) /Blood Urea Nitrogen (BUN) (mg/dl)
Cow urine (mg/dl)	69 ± 1.87
Cow dung (%)	2.51 ± 0.24
Compost (%)	2.25 ± 0.03
Poultry litter (%)	1.98 ± 0.12
UTR (%)	1.28 ± 0.26
NPK (%)	1.11 ± 0.43

## 3 Data Description

Data were collected on growth performance of marine *Chlorella* sp. cultured in different cost-effective media including cow dung, cow urine, poultry litter, compost, NPK, UTR [1]. Onset of stationary phase (6–9 days) varied among the cow dung, poultry litter, compost, NPK, UTR at 5 mg of concentration (5 mg of media diluted in 350 ml of sea water) and for cow urine at 5 µL of concentration (5 µL of this media diluted in 350 ml of sea water). In cow dung and UTR media stationary phase has accelerated in 6 days, Compost in 7 days, Conway, NPK and poultry litter in 8 days and cow urine in 9 days (Table 2).

**Table 2:** The growth in terms of cell density (cells/mL × 10^6)^ of *Chlorella* sp. in different media at 5 mg of concentration. Significant variations (p < 0.05) were found among different media (Values are means ± SD).

Days	Conway (Mean ± SD)	Cow urine (Mean ± SD)	Cow dung (Mean ± SD)	Compost (Mean ± SD)	UTR (Mean ± SD)	NPK (Mean ± SD)	Poultry litter (Mean ± SD)
0	0.14 ± 0.026	0.3 ± 0.173	0.12 ± 0.026	0.0675 ± 0.005	0.081 ± 0.002	0.06 ± 0.026	0.183 ± 0.005
1	0.18 ± 0.02	0.105 ± 0.009	0.135 ± 0.007	0.168 ± 0.015	0.238 ± 0.023	0.258 ± 0.02	0.183 ± 0.006
2	0.21 ± 0.017	0.163 ± 0.028	0.26 ± 0.026	0.265 ± 0.018	0.25 ± 0.026	0.592 ± 0.032	0.35 ± 0.035
3	0.34 ± 0.036	0.54 ± 0.026	0.593 ± 0.091	0.43 ± 0.036	0.223 ± 0.024	0.318 ± 0.045	0.477 ± 0.011
4	0.518 ± 0.039	0.18 ± 0.02	0.877 ± 0.075	0.195 ± 0.007	0.267 ± 0.021	0.225 ± 0.011	0.4 ± 0.265
5	1.09 ± 0.121	0.745 ± 0.069	0.377 ± 0.031	0.428 ± 0.04	0.267 ± 0.021	1.86 ± 0.118	0.445 ± 0.008
6	2.03 ± 0.036	1.1 ± 0.265	1.12 ± 0.123	0.585 ± 0.014	0.283 ± 0.006	2.29 ± 0.243	1.06 ± 0.062
7	6.87 ± 0.145	0.925 ± 0.125	0.65 ± 0.066	0.8 ± 0.265	0.143 ± 0.006	2.88 ± 0.115	1.07 ± 0.046
8	11.3 ± 0.755	0.865 ± 0.091	0.468 ± 0.087	0.435 ± 0.063	0.0525 ± 0.008	3.27 ± 0.154	1.28 ± 0.056
9	10.6 ± 1.179	1.51 ± 0.165	0.22 ± 0.036	0.255 ± 0.023	0.05 ± 0.01	2.11 ± 0.191	0.648 ± 0.014
10	5.78 ± 0.372	0.8 ± 0.265					

**Fig. 1:**
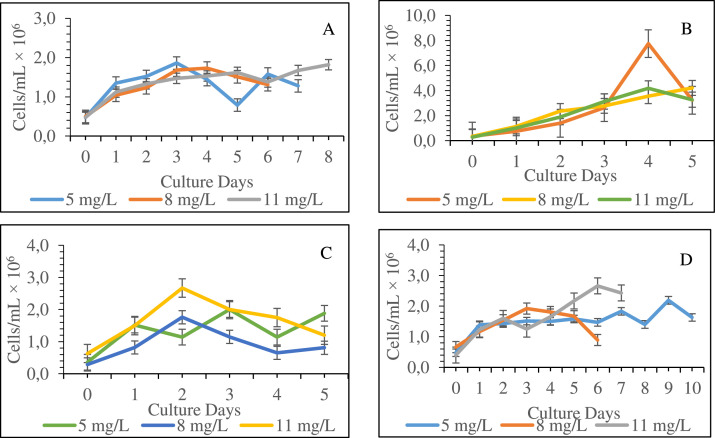
Growth curve in terms of cell density (cells/mL × 10^6^) of marine microalgae *Chlorella* sp. cultured in cow urine (A), NPK (B), cow dung (C), and poultry litter (D). All the data of cell density significantly (p < 0.05) varied among different concentrations of similar media (Values are means  ±  SD).

Poultry litter, cow urine, cow dung and NPK media were screened after culturing the *Chlorella* species in six different media with 5 mg and 5 µL (cow urine) of concentration along with conway media and set another experiment with these four media with 5 mg, 8 mg, and 11 mg of concentration to observe the growth curve of the microalgae. Here, 5 mg of concentration was used as control and to compare the growth in 8 mg, and 11 mg respectively. Since the cell density in compost and UTR media at 5 mg concentration was significantly lower than that in other media, these media were discarded. Cow urine media had a lower cell density than other media for each concentration. Growth of *Chlorella* in NPK with 8mg and 11mg of concentration showed highest cell numbers on 6^th^ and 5^th^ day respectively with 4.21 × 10^6^ and 4.18 × 10^6^ cells/mL. Cow dung with 11 mg of concentration showed 2.67 × 10^6^ cells/mL which is about 1.5 times higher to maximum growth in the same concentration of poultry litter on 3^rd^ day. Growth curves of the marine microalgae *Chlorella* species, cultured in four different media at 5 mg, 8 mg and 11 mg concentration are shown in this data (Fig. 1).

**Fig. 2:**
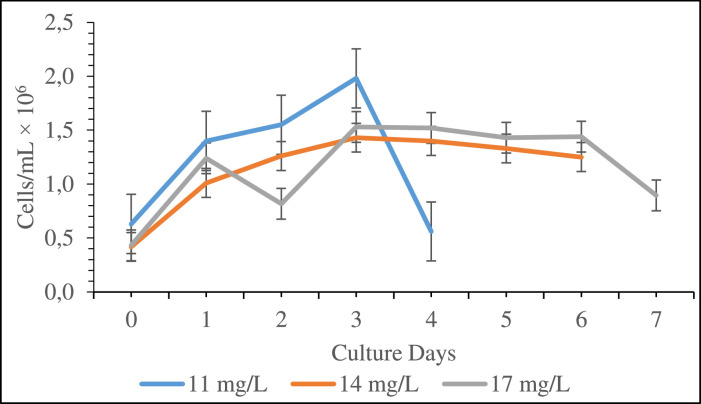
Growth curve in terms of cell density (cells/mL × 10^6^) of marine microalgae *Chlorella* sp. cultured in different concentration of cow dung. All the values of cell density significantly (p < 0.05) varied among three different concentrations of cow dung media. Values are means ± SD of triplicates measurements.

*Chlorella* sp. had a higher cell density in NPK media than in other media, however this media was discarded since it is inorganic and costly. The focus of the subsequent experiment was placed on cow dung media because cow urine media had a low cell density and poultry litter media had a prolonged stationary phase. The data represented in the following graph (Fig. 2) shows that the cell number was still greater in the 11 mg of cow dung concentration when compared to the 14 mg and 17 mg of cow dung concentrations.

## 4 Experimental Design, Materials and Methods

### 4.1 Collection and maintenance of *Chlorella* sp. culture

Pure isolates of the selected microalgae, *Chlorella* sp., were collected from the laboratory of the Live Feed Research Corner, Faculty of Fisheries, Chattogram Veterinary and Animal Sciences University. The pure samples were cultured at 25 ppt salinity in conway culture medium. Stock was scaled up, and then sub cultured for growth curve determination.

### 4.2 Experimental design

Some locally available organic (cow dung, cow urine, poultry litter, compost) and inorganic (UTR, NPK) media were collected for the culture of marine *Chlorella* sp. Initial selection of four media, including cow dung, cow urine, poultry litter, and NPK, was made based on cell density and the onset of stationary phase. After culturing at different concentrations, suitable media for *Chlorella* sp. culture was chosen.

#### 4.2.1 Nitrogen content determination

Nitrogen is an important nutrient for microalgal growth because the formation of amino acids, proteins, coenzymes, enzymes, chloroplasts, and so on is linked to nitrogen assimilation [5]. Microalgae can use a variety of nitrogen sources, including ammonia, nitrite, nitrate, and urea. The nitrogen content of cow urine media was determined by photometric method [6]. Blood Urea Nitrogen (BUN) of cow urine was found 69 mg/dl. Protein content of cow dung, compost, poultry litter, UTR, and NPK was determined [7]. The general conversion factor was used to determine these media's nitrogen content [8]. Cow dung, compost, poultry litter, UTR, and NPK had total nitrogen content of 2.51%, 2.25%, 1.98%, 1.28%, and 1.11%, respectively.

### 4.3 Media preparation

#### 4.3.1 Conway media

Micronutrients, trace metal solution, and vitamin D are all included in conway medium [9]. *Chlorella* sp. culture was carried out in pure conway medium; the following table shows the amounts of various constituents. 1 mL of solution A, 0.5 mL of solution B, and 0.1 mL of solution C were mixed with 28-30 g/L autoclaved seawater to make 1L of conway media (Table. 3).

**Table 3:** Constituents of microalgae culture medium.

(A) Main Mineral Solution	
Names of Chemicals	Quantity
NaNOз/KNOз	100.00 g/116.00 g
Disodium EDTA (C_10_ H_16_N_2_O_8)_	45.00 g
H_3_BO_3_	33.60 g
NaH_2_PO_4_.4H_2_O	20.00 g
FeCL_3_.6H_2_O	1.30 g
MnCL_2_.4H_2_O	0.36 g
Trace metal solution	1.00 mL
Dissolving in deionized/distilled water and make the volume 1 L	
(B) Trace Metal Solution	

Names of Chemicals	Quantity

ZnCl_2_	2.10 g
CoCl_3_.6H_2_O	2.00 g
(NH_4_)_6_MO_7_O_2_.4H_2_O	0.90 g
CuSO_4_.5H_2_O	2.00 g
Dissolving in deionized/distilled water and make the volume 1 L	
(C) Vitamin	

Names of Chemicals	Quantity

Thiamine, B1	0.20 g
Cyanocobalamin, B12	0.01 g
Dissolved in deionized/distilled water and make the volume 100 mL	

#### 4.3.2 Inorganic media

##### 4.3.2.1 Urea, TSP, and red potash (UTR)

UTR fertilizers were collected in solid form. These components were then mixed in a similar weight ratio (U: T: R = 1:1:1). The solid mixture of these components was mixed with 25 ppt sea water to make a liquid solution of this media, which was autoclaved at 121 °C for 15 min. The solution of this media was used at the required concentration with autoclaved sea water.

##### 4.3.2.2 Nitrogen, phosphorus, and potassium (NPK)

NPK fertilizer is also available in solid powder form. The solid fertilizer was mixed with 25 ppt sea water at the required concentration to make a liquid NPK fertilizer solution, which was autoclaved at 121 °C for 15 min. and used as culture media.

#### 4.3.3 Organic media

##### 4.3.3.1 Cow urine

Cow urine was collected from the Chattogram Veterinary and Animal Sciences University's cattle farm. After autoclaving it for 15  min at 121 °C, it was diluted with 25 ppt sea water to prepare the required concentration of culture media.

##### 4.3.3.2 Cow dung, compost, poultry litter

Raw cow dung collected from cattle farm in the same way that cow urine was collected, compost fertilizer was purchased in both wet and raw form, and poultry litter was collected from poultry farm in raw form. All of these were sun-dried and blended to create a dry powder form. The powder was then poured into 25 ppt sea water, which was vortexed (using Vortex Mixer, VM-10, Witeg, Germany) for 2-5 min and sonicated (using Ultrasonic Bath, Model-621.06.010, ISOLAB, Germany) at 37 kHz, 50 °C for 15  min to ensure proper mixing. Following sonication, the liquid solution was filtered (47 mm Whatman® GF/C glass microfiber filter papers) to remove the debris. Finally, the liquid portion of the solution was autoclaved at 121 °C for 15  min before being diluted with 25 ppt sea water to prepare the required concentration of culture media. The concentrations of Total Nitrogent (TN) and Blood Urea Nitrogen (BUN) remained unchanged after autoclaving the above described media.

### 4.4 Availability and approximate cost of each medium

Organic media including cow dung, cow urine, compost, and poultry litter were collected free of cost from the Chattogram Veterinary and Animal Sciences University's farm. On the other hand, inorganic media, which included NPK for 5.42 USD/Kg (4.99 Euro/Kg) and UTR at 0.99 USD/Kg (0.91 Euro/Kg), were purchased from Chattogram's local market. For preparation of commercial conway media, all the required chemicals were ordered and the media was finally prepared in the lab following the standard protocol. Traditionally used commercial media conway costed 14.08 USD/Litre (12.97 Euro/Litre).

### 4.5 Determination of growth curve

Data of the growth curves of *Chlorella* sp. were determined to screen the low-cost media. A total of 350 mL of culture volume was maintained in a sterile 500 mL borosilicate Erlenmeyer flask for each species with three replicates. Stock culture was added at a rate of 4% to the 350 mL of culture media. The culture was continued up to the death phase. Depending on culture medium, onset of stationary phase varied. Water salinity was maintained 25 ppt, and gentle aeration was provided continuously. Growth curve was determined on basis of cell density (cells/mL).

#### 4.5.1 Cell density

During the data collection for the growth curve, *Chlorella* cells were counted using a hemacytometer on a daily basis. Before filling the chambers with culture samples, the hemacytometer and its cover slip (Bright-line upgraded Neubauer hemacytometer, 0.0025 mm^2^, 0.1 mm deep chambers, Assistent, Germany) were cleaned using Milli-Q water (Millipore Corp) to make sure that it was free of dust, lint and grease. Subsequently, a little drop of properly mixed sample was introduced into the counting chamber. In the meantime, the pipette was kept at an angle until a small drop of sample appeared at its tip. The drop was then positioned so that the capillary action was between the cover glass and the counting chamber base. No bubbles appeared, and for better counting, the cells were let 3 to 5  min to settle. Under low power magnification (4x and 10x) of the microscope (Nikon E600), the evenness of cell distribution was examined. Under 40x magnification, cells were counted in the hemacytometer's two chambers. The following formula was used for the cell density calculation [10]:

$$\text{Cell}\;\text{count}\;\text{calculation}\;\left(\text{cell}/\text{mL}\right)\;\text{for}\;25\;\text{squares}=\;\frac{\text{Total}\;\text{number}\;\text{of}\;\text{cells}\;\text{counted}}{50\times 4}\times 10^{6}$$

In this equation, 50 stood for the 50 squares within each of the two hemacytometer chambers, and 4 × 10^−6^ for the volume of samples spread among the tiny square areas, which was equal to 0.004 mm^3^ (0.2 mm × 0.2 mm × 0.1 mm), expressed in cm^3^ (mL).

### 4.6 Quality assurance and quality control

Every instrument was calibrated in compliance with the relevant standard operating procedures (SOPs) and the manufacturer's instructions. Regular calibrations were performed on pipettes, balances, and other measuring instruments. Before starting each test, the bottle labels were double checked. The tests that were conducted yielded consistent results. Apparatus used for analysis was rinsed three times with distilled water and covered with aluminium foil before and after use. A blank was analyzed with every set of samples in nitrogen content determination. Triplicates were counted for cell count.

All the glassware, sea water, all media diluted in 25 ppt water, cotton ball etc. were properly sterilized. In the biological safety cabinet, all the equipment was UV treated prior to the inoculation of microalgae samples in various media. Before and after use, the cabinet was cleaned with 80% ethanol. To conduct the experiment, a healthy and contamination-free *Chlorella* sp. sample was used. When working in the laboratory, safety precautions were taken, such as wearing the appropriate lab coat, mask, and hand gloves.

All media, except conway, were autoclaved and then streaked onto agar plates (MacConkey Agar, Salmonella Shigella Agar, Thiosulfate-Citrate-Bile-Salt Sucrose Agar, Mannitol Salt Agar, Nutrient Agar, and Pseudomonas Agar). The plates were then kept in an incubator at 37 °C. No bacterial colony was observed in any media.

### 4.7 Statistical analysis

One-way analysis of variance (ANOVA) was used to test all the data. Significant differences in means among groups and at different time points were evaluated by Tukey's test. Statistical analyses were accomplished using the Statistical Analysis System (SAS 9.2) computer software. The percent differences between groups were calculated and considered significant at the level of p < 0.05.

## Limitations

Not applicable.

## CRediT authorship contribution statement

**Trina Das:** Conceptualization, Data curation, Writing – original draft. **Sifatun Nur:** Data curation, Formal analysis. **Mohammad Ekramul Haque:** Data curation, Formal analysis, Writing – review & editing. **Mahima Ranjan Acharjee:** Data curation, Formal analysis, Writing – review & editing. **Subeda Newase:** Data curation, Formal analysis. **Sadia Afrin:** Data curation, Formal analysis. **Helena Khatoon:** Conceptualization, Funding acquisition, Supervision, Resources, Validation, Writing – review & editing.

## Data Availability

Data on growth performance of marine Chlorella sp. cultured in different cost-effective media (Original data) (Mendeley Data).
